# Supplementary material for: Prevalence of loss-of-function, gain-of-function and dominant-negative mechanisms across genetic disease phenotypes
Source: Nat Commun. 2025 Sep 25;16:8392. doi: 10.1038/s41467-025-63234-3 (PMC12462468; doi:10.1038/s41467-025-63234-3)
Supplement: Supplementary file 1 — Supplementary Information [file 41467_2025_63234_MOESM1_ESM.pdf]

Supplementary Information for

Prevalence of loss-of-function, gain-of-function and dominant-negative mechanisms across genetic disease phenotypes

Mihaly Badonyi[1*], Joseph A Marsh[1*]

[1] MRC Human Genetics Unit, Institute of Genetics and Cancer, University of Edinburgh, Edinburgh, UK

*correspondence to joseph.marsh [at] ed.ac.uk; mihaly.badonyi [at] ed.ac.uk

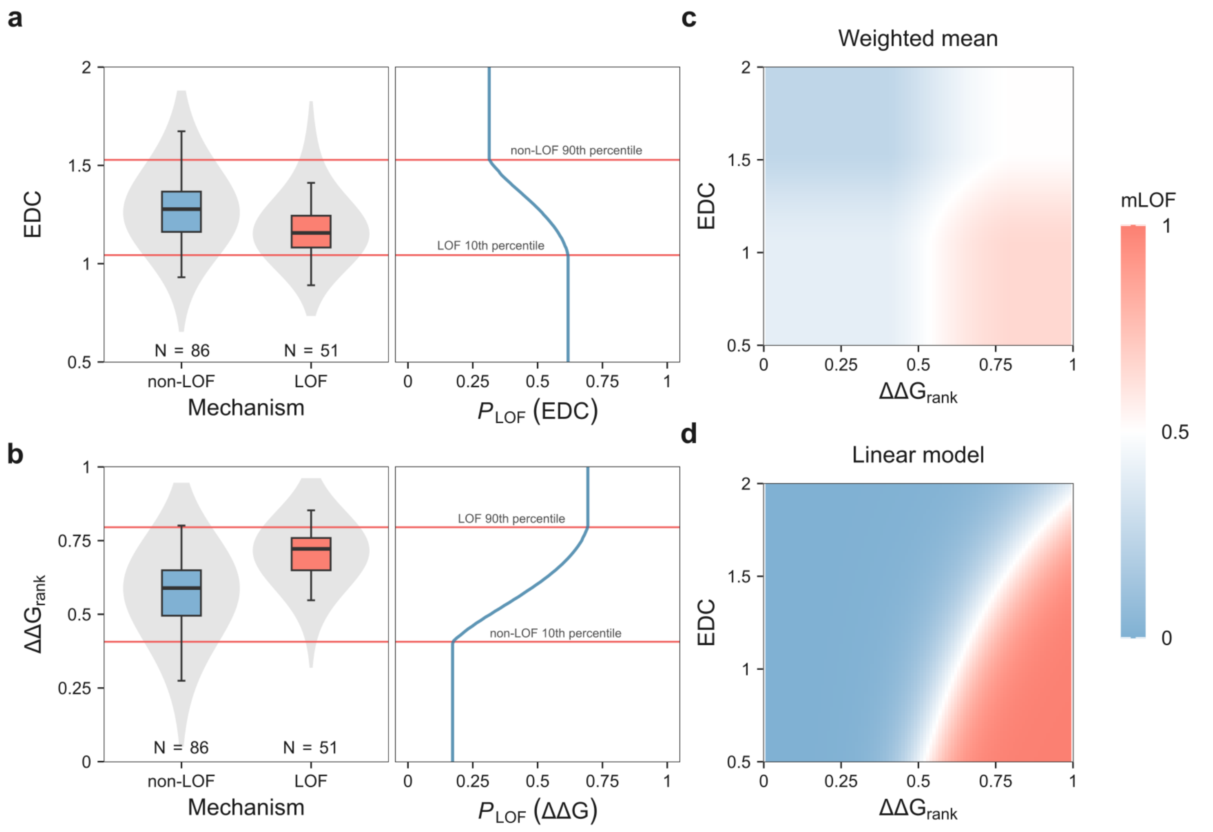

Supplementary Fig. 1. An empirical distribution-based approach to derive the mLOF score. a: EDC and **b**: $\Delta\Delta G_{rank}$ distributions of genes primarily associated with missense LOF and non-LOF mechanisms. N represents the number of proteins in each group. The plot on the right shows the distribution of estimated LOF probability given the observed EDC or $\Delta\Delta G_{rank}$ value (P_{LOF}(EDC) and $P_{LOF}(\Delta\Delta G)$, respectively). Red lines represent 10th and 90th percentile caps. **c**: Landscape of combined probabilities (mLOF score) for the expected range of EDC and $\Delta\Delta G_{rank}$ values with the weighted mean method (used in this study), using a global median weight of 1.47:1 (EDC:$\Delta\Delta G_{rank}$) for the purpose of this visualisation. **d**: Landscape of mLOF scores with the generalised linear model (not used in this study). Source data are provided as a Source Data file.

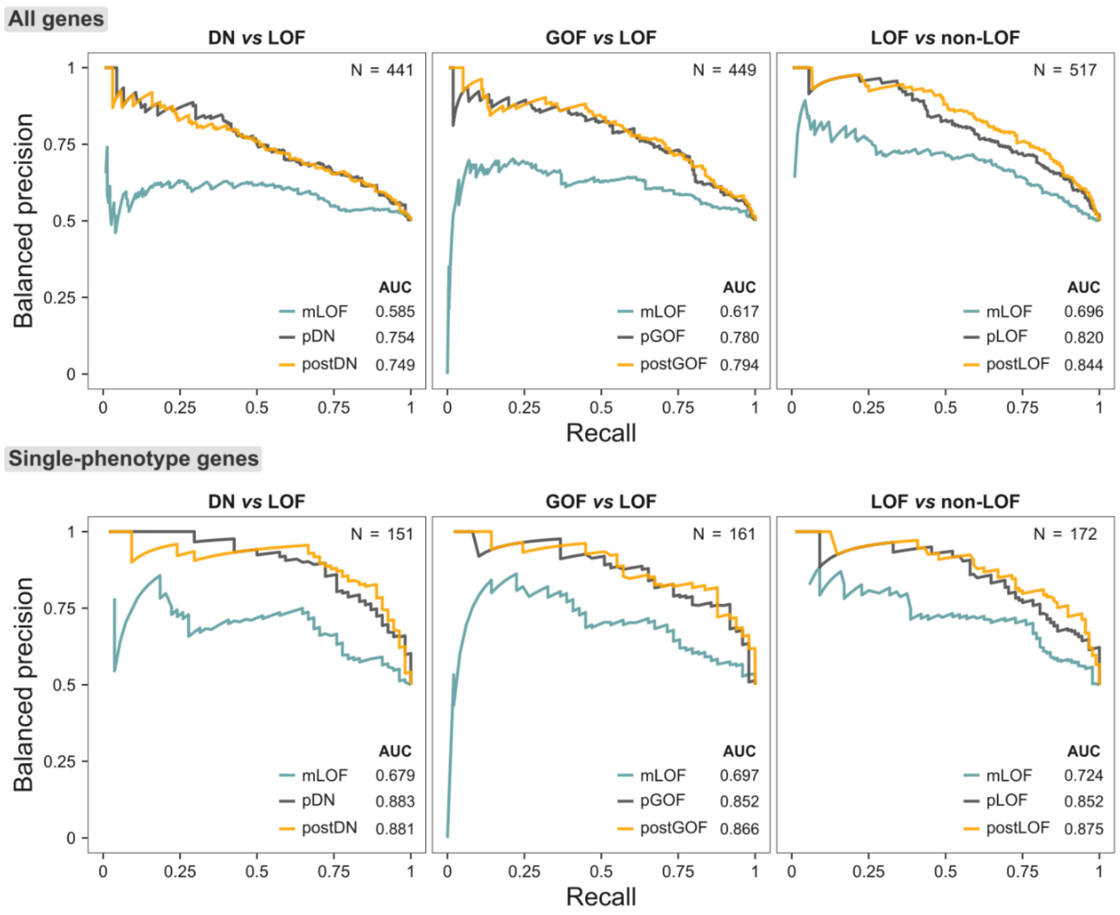

Supplementary Fig. 2. Balanced precision-recall curves, related to Fig. 1b. Phenotype-level balanced precision-recall curves and area under the curve (AUC) values of the mLOF score, the prior mechanism probability for the gene (one of pDN/GOF/LOF), and the posterior mechanism-specific scores (one of postDN/GOF/LOF) across the binary class pairs used to construct the priors, split into all genes and single-phenotype genes. N is the number of genes in each group. Source data are provided as a Source Data file.

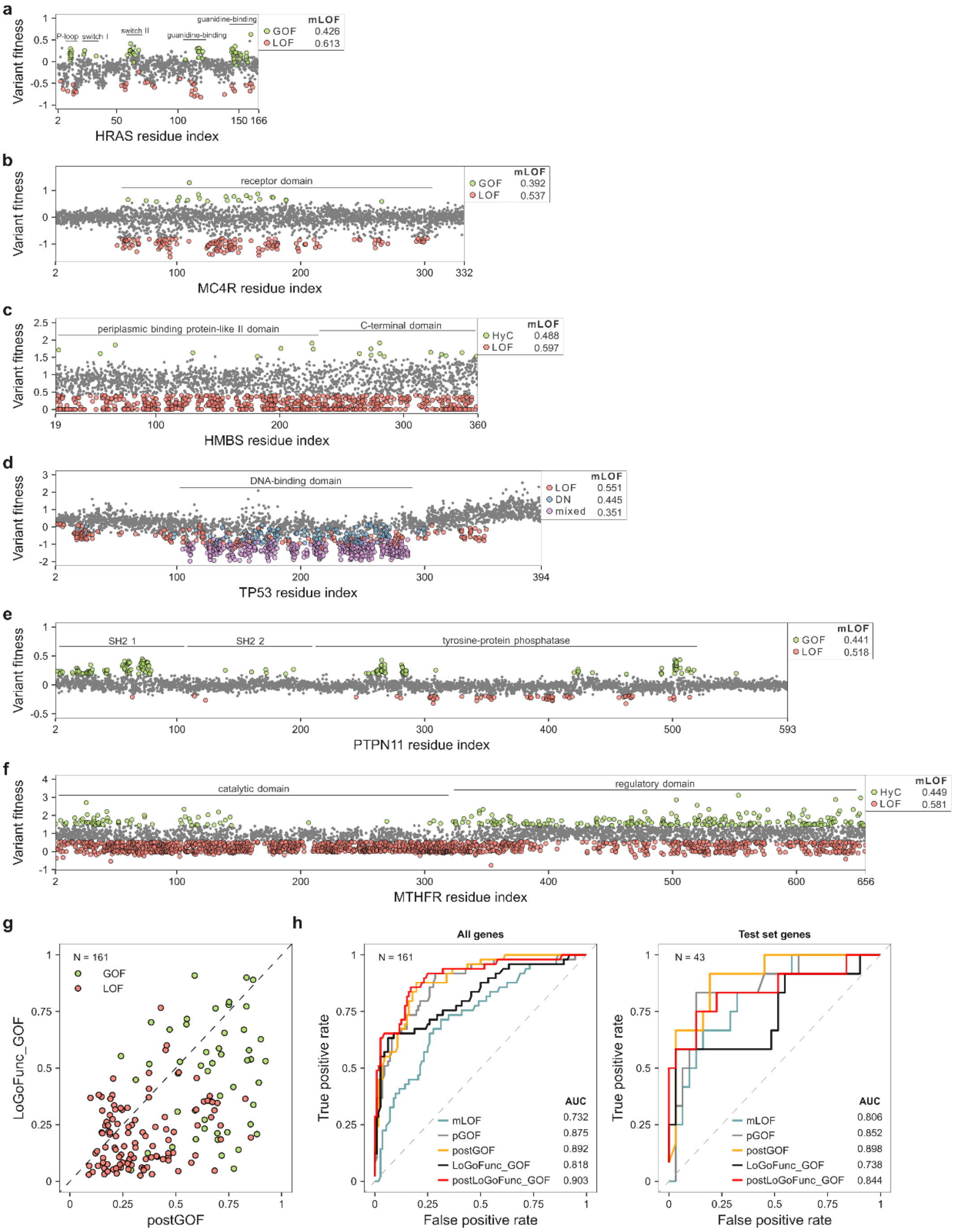

Supplementary Fig. 3. Comparison of the mLOF score with functional assay results and computational predictions of GOF variants. a-f: Analysis of multiplexed assays of variant effect (MAVEs) for HRAS, MC4R, HMBS, TP53, PTPN11 and MTHFR. Each data point represents the measured fitness of the variant (*y* axis) at the given residue position (*x* axis). The variants are coloured according to their functional effect category: loss of function (LOF), gain of function (GOF), hyper-complementing (HyC), dominant-negative (DN), and in the case of TP53, hybrid LOF/DN variants (mixed). The mLOF score for the different functional categories is shown on the right of the plots. **g**: Scatter plot of postGOF *vs* average LoGoFunc_GOF probability for phenotypes in single-phenotype AD genes, coloured by their reported GOF/LOF disease mechanisms. N is the number of genes. **h**: Receiver operating characteristic (ROC) curves and area under the curve (AUC) values of the mLOF score, pGOF, postGOF, the average LoGoFunc_GOF probability, and postLoGoFunc_GOF for the phenotypes. N is the number of genes. Source data are provided as a Source Data file.

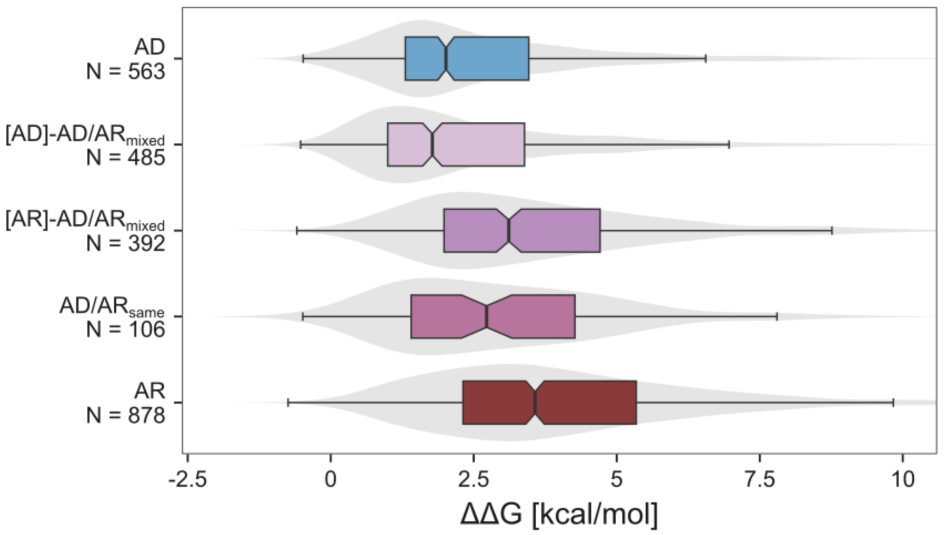

Supplementary Fig. 4. Supplemental analysis to Fig. 3a using raw ΔΔG values and including all inheritance groups. Source data are provided as a Source Data file.

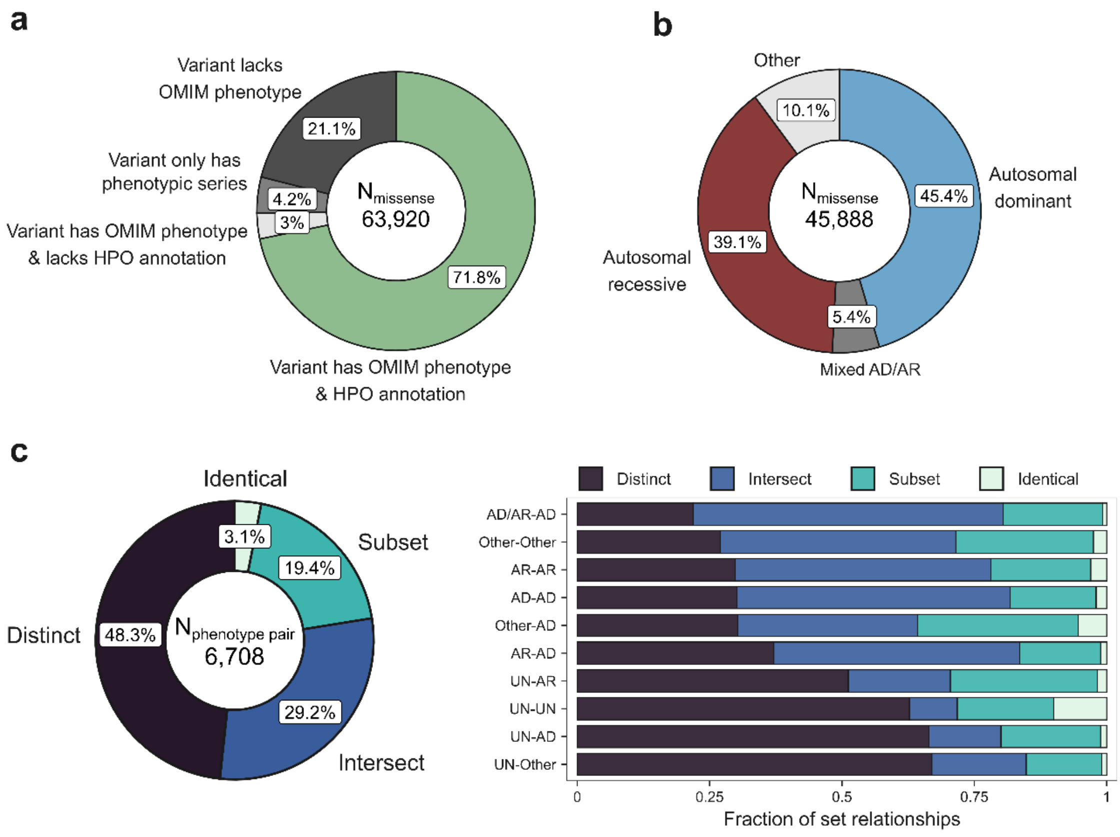

Supplementary Fig. 5. Missense variant statistics in terms of phenotype composition and overlap. a: The composition of available OMIM phenotype annotations for pathogenic missense variants after cross-referencing with HPO data. **b:** Inheritance composition of variants with OMIM phenotype and HPO annotation. **c:** As disease phenotypes may share ClinVar missense variants, these can be grouped by their set relationships. This panel shows the distribution of within-gene inheritance pairs based on shared variants, with phenotype pairs classified as *distinct*, *intersecting*, *subset*, or *identical*. Proportions are shown both overall and stratified by inheritance mode. While our analyses focus on the distinct and intersecting categories, the full distribution is displayed here to provide context. Only inheritance pairs with at least 100 shared variants are included. Source data are provided as a Source Data file.
