# Supplementary material for: Prevalence of loss-of-function, gain-of-function and dominant-negative mechanisms across genetic disease phenotypes
Source: Nat Commun. 2025 Sep 25;16:8392. doi: 10.1038/s41467-025-63234-3 (PMC12462468; doi:10.1038/s41467-025-63234-3)
Supplement: Supplementary file 2 — Description of Additional Supplementary Files [file 41467_2025_63234_MOESM2_ESM.pdf]

Description of Additional Supplementary Files

File Name: Supplementary Data 1

Description: The data contains MIM identifiers, disease names, EDC and ΔΔGrank values, mLOF scores, and the mechanism-specific posterior scores for all analysed disease phenotypes.
